# Fear of Missing Out, Social Media Addiction, and Personality Traits Among Nursing Students: Cross-Sectional Study

**DOI:** 10.2196/71502

**Published:** 2025-05-29

**Authors:** Amira Alshowkan, Emad Shdaifat

**Affiliations:** 1 College of Nursing, Imam Abdulrahman Bin Faisal University, King Faisal Street, PO Box 1982, Dammam, Saudi Arabia, 966 544312234

**Keywords:** social media, personality, fear, neuroticism, conscientiousness, students, nursing

## Abstract

**Background:**

The growing use of social media has created concerns about addiction, and thus, it is necessary to explore how personality traits and fear of missing out (FOMO) can be utilized to predict social media addiction (SMA).

**Objectives:**

The purpose of this study was to investigate the connection between personality traits, FOMO, and SMA in university students in Saudi Arabia.

**Methods:**

In this cross-sectional study, data were collected from nursing students using the shortened version of the big five inventory, fear of missing out scale, and SMA scale from May to September 2024.

**Results:**

The study achieved a response rate of 66.7% (414/620), finally including a total of 411 participants. The majority of participants (247/411, 60.1%) had low FOMO scores, while SMA scores showed a different pattern, with a larger proportion (261/411, 63.5%) of participants scoring in the moderate range. In terms of gender differences, male participants exhibited higher levels of FOMO (*t*=3.86, *P*<.001) and SMA (*t*=2.51, *P*=.013) compared to female participants. Additionally, male participants scored higher in neuroticism (*t*=3.30, *P*=.001) and openness (*t*=1.98, *P*=.048). Regression analysis revealed that both conscientiousness (*β*=.357, *P*<.01) and FOMO (*β*=.213, *P*<.01) positively predicted SMA, while neuroticism (*β*=−.223, *P*<.01) and being female (*β*=-.098, *P*<.05) were associated with lower levels of addiction. The resulting model accounted for 35.8% of the variance.

**Conclusions:**

The study provides evidence that conscientiousness and FOMO are positive predictors of SMA, while neuroticism is negatively correlated with it. Moreover, male participants exhibited higher levels of both FOMO and SMA in comparison to female participants. These findings emphasize the impact of personality traits and FOMO on SMA among university students.

## Introduction

The proliferation of social media has revolutionized the way people connect and consume information. Yet, alongside the advantages of social media, several issues have been raised concerning its potential negative effects on a person’s well-being [1]. Social media addiction (SMA), also described as problematic social media use, is exemplified by extreme and compulsive usage of social media that negatively impacts an individual’s life. Like other forms of behavioral addictions, SMA enmeshes a loss of control over one’s usage, social media activities preoccupation, and continued engagement despite adverse outcomes. Numerous studies have investigated the determinants and outcomes of SMA [2-5]. Factors such as personality traits, social influences, and fulfillment of psychological needs have been implicated in the development and maintenance of SMA [2,3]. Moreover, SMA has been associated with a range of negative outcomes, including impaired academic performance, diminished real-life social relationships, and increased risk of mental health problems such as depression and anxiety [4,5].

Fear of missing out (FOMO) refers to the apprehension or anxiety individuals experience when they believe that others are engaging in enjoyable activities or experiences from which they are excluded [6,7]. Research indicates that people with elevated FOMO have a high probability of engaging in excessive social media use, experience higher levels of stress and anxiety, and report lower levels of life satisfaction and well-being [7,8]. Additionally, FOMO has been linked to problematic behaviors such as compulsive smartphone checking, which can further exacerbate feelings of anxiety and dissatisfaction [9].

Personality traits have a considerable role in individuals’ susceptibility to FOMO and SMA. Research has identified several personality traits that may contribute to these phenomena [7]. For example, individuals high in neuroticism, considered by tendencies toward anxiety and other negative emotions, may be more prone to experiencing FOMO and engaging in excessive social media use as a means of stress-coping strategy [7,9]. Similarly, individuals with high levels of extraversion may also be at increased risk of developing problematic patterns of social media use, as they may seek social validation and affirmation through online interactions [3]. The interplay between FOMO, SMA, and personality traits is complex and multifaceted. Personality traits may influence individuals’ susceptibility to FOMO and SMA, while these phenomena, in turn, may exacerbate underlying personality vulnerabilities. For instance, people high in neuroticism may be particularly susceptible to developing SMA as a means of alleviating feelings of anxiety and insecurity correlated with FOMO [9]. Few studies focus on personality types and their relation to FOMO [7,10]. Research suggests that neuroticism is positively correlated with FOMO, as people with higher levels of neuroticism have a high probability of experiencing fear, insecurity, and anxiety related to missing out on rewarding experiences [7]. While extraversion is typically associated with sociability and outgoing behavior, it can also be linked to FOMO, particularly in the context of social comparison and the desire for social validation. Extraverted individuals may be more likely to engage in social media use in order to stay connected with others and seek external validation, leading to higher levels of FOMO [10].

Openness to experience reflects individuals’ receptivity to new ideas, experiences, and perspectives. While research on the relationship between openness and FOMO is limited, some studies suggest that individuals high in openness may be more motivated to seek out novel experiences and social interactions, potentially increasing their susceptibility to FOMO [11]. Conscientiousness is characterized by self-discipline, organization, and goal-directed behavior. Although less studied than other personality traits, conscientiousness may play a role in individuals’ susceptibility to FOMO, particularly concerning academic and professional goals. High levels of conscientiousness may lead individuals to prioritize staying informed and connected with others, contributing to higher levels of FOMO [10]. Furthermore, research suggests that individuals may use social media as a means of seeking validation and approval from others, leading to heightened feelings of anxiety and insecurity related to missing out on social experiences [11].

The negative consequences of SMA and FOMO on the mental and physical health of university students were reported; studies have shown that the extreme use of social media is linked with symptoms of depression and poor physical activity, exacerbating mental illness [12]. Moreover, SMA has been associated with body image problems, because students compare themselves to unrealistic standards, which results in body dissatisfaction, and therefore, irrational attitudes [13,14]. SMA additionally warps weight perception, which is concerning for the reason that it affects students’ lifestyle [15]. Furthermore, continuous exposure to social media may impact health behaviors, for instance, eating habits, which can lead to negative health effects [16]. Thus, the interaction of SMA and FOMO among university students may destroy physical and psychological well-being.

Therefore, it is significant to explore SMA, FOMO, and personality traits among university students, as social media usage is progressively becoming more widespread and might negatively affect mental health, academic performance, and well-being. University students, as they are under the stress of academic and social comparison, are more vulnerable to social media impacts. Understanding how personality influences social media use can help identify students at risk of addiction and the ensuing mental challenges. It is vital to conduct this research to recommend interventions that promote healthier digital habits and improve students’ overall well-being. Moreover, among nursing students in Saudi Arabia, little is known about the FOMO and SMA. Thus, this study aims to explore nursing students’ personality traits and their relation to the FOMO and SMA in the era of rapid technology.

## Methods

### Site, Setting, and Design

A cross-sectional study was conducted at the College of Nursing, Imam Abdulrahman Bin Faisal University, located in the Eastern Province of Saudi Arabia, from May to September 2024.

### Sampling

The target population for this study comprised students enrolled in the College of Nursing. The inclusion criteria consisted of nursing students from the first to the fifth year. Students who had postponed their courses or withdrawn from the nursing college were excluded. A convenience sampling method was employed due to practical constraints, including limited access to a comprehensive student list and time restrictions. Given that the study aimed to explore associations rather than generalize findings to the entire population, nonprobability sampling was deemed appropriate. While probabilistic sampling enhances generalizability, its implementation was not feasible within the context of this research. The sample size was calculated based on the Raosoft calculator [17], based on the total population of 1129 people, 95% confidence level, and 5% margin. The sample of 287 nursing students was considered representative. Although the calculated sample size was 287, a total of 411 students responded to the survey, and all responses were valid.

Students were contacted via their university email by the registration office. A research pack was sent, which included an information sheet with detailed study information, along with a web-link and barcode to access the study questionnaires. A follow-up email was sent 2 weeks later to remind students to complete the questionnaires. The questionnaires took approximately 30‐45 minutes to complete.

### Study Tools

#### Fear of Missing Out

The FOMO scale [7] is a self-report instrument designed to measure individuals’ tendencies to experience FOMO on rewarding experiences. The questionnaire consists of 10 items, each assessing different aspects of FOMO. Respondents rate their agreement with each item on a scale, typically ranging from 1 (strongly disagree) to 7 (strongly agree). Example items include “I fear others have more rewarding experiences than me” and “I fear my friends have more rewarding experiences than me.” The Cronbach α coefficient for the FOMO scale has been reported to be around .85, indicating high internal consistency among its items. The scale validity and reliability were measured and confirmed to be appropriate for use in Arabic culture [18]. For this study, the Cronbach α coefficient for the FOMO construct was calculated to be 0.757, while the McDonald ω coefficient was determined to be 0.744.

#### Personality Traits

The big five personality traits were assessed using a shortened version of the big five inventory, consisting of 44 Likert-scale items. Participants rated their agreement on a scale from 1 (strongly disagree) to 5 (strongly agree). This inventory encompasses five subscales: conscientiousness, extraversion, agreeableness, openness, and neuroticism [19]. Example items include “I see myself as someone who is talkative,” “I see myself as someone who tends to find fault with others,” and “I see myself as someone who does a thorough job.” Validity and reliability assessments for the Arabic version were conducted, revealing Cronbach α coefficients ranging from 0.84 to 0.68 across the subscales [20]. The Cronbach α for the totality of personality traits was calculated to be 0.704, while the McDonald ω was determined to be 0.653.

In addition, the students’ sociodemographic data were collected using a form developed by the researchers, which included information such as age, gender, and marital status.

### SMA

The social media addiction scale [18] was employed to assess social media usage. This scale, derived from the internet addiction test [21], comprised 14 items tailored to gauge SMA specifically. Respondents rated these items on a 5-point Likert scale, ranging from strongly agree to strongly disagree, with corresponding scores of 5 to 1. Example items include “I often find myself using social media longer than intended” and “I often find life to be boring without social media.” The validity and reliability of the social media addiction scale were examined and proven appropriate for use in Arabic culture [18]. The Cronbach α coefficient for the SMA was 0.837, while the McDonald ω coefficient was 0.841.

### Ethical Considerations

The study received ethical approval from the Institutional Review Board (IRB) at Imam Abdulrahman Bin Faisal University, under approval number IRB-2024–04-475. The IRB endorsed the study procedures and surveys prior to the initiation of participant recruitment. The participants consisted of nursing students from the College of Nursing at Imam Abdulrahman Bin Faisal University. Prior to participation, students were provided with a comprehensive information sheet that outlined the voluntary nature of the study, as well as their right to withdraw at any time without compromising their academic standing or rights. Participants received a detailed explanation of the study, including its associated risks and benefits. They were assured that their confidentiality and privacy would be upheld in accordance with the study’s established guidelines. The information sheet also delineated the research objectives, significance, and potential benefits of the study. Informed consent was obtained from all study participants.

### Data Analysis

Data were collected and analyzed utilizing SPSS (version 22; IBM Corp) and Microsoft Excel. Categorical variables were assessed through frequencies and percentages, whereas continuous variables were described using means and standard deviations. The reliability of the scales was evaluated using Cronbach α coefficients. Differences between groups and relationships among variables were analyzed employing *t* tests. Correlation analysis was conducted to examine relationships between various variables. To predict SMA scores, a multiple stepwise regression analysis was conducted, incorporating demographic variables and personality traits, specifically extraversion, agreeableness, conscientiousness, and neuroticism, alongside the FOMO. A *P* value of less than .05 was deemed statistically significant.

Data cleaning and screening were performed utilizing SPSS and Microsoft Excel. The response rate yielded a 66.7% rate of participation, with a total of 414 individuals involved in the study. One participant was eliminated due to zero variance, indicating a lack of engagement, while 2 participants were excluded as outliers based on Mahalanobis distance [22]. The individual personality trait α values were as follows: 0.640 for extraversion, 0.646 for agreeableness, 0.735 for conscientiousness, 0.819 for neuroticism, and 0.614 for openness. Reverse coding was applied where necessary. Both FOMO and SMA scales were categorized into three levels based on the calculated interval of (5 - 1)/3=1.33 [23]. The ranges were delineated as follows: a low score was defined as falling between 1 and 2.33, a moderate score was classified as ranging from 2.34 to 3.67, and a high score was indicated as any value exceeding 3.67.

## Results

Table 1 presents the demographic characteristics of the 411 study participants. A significant majority of the participants were female participants (302/411, 73.5%), with a predominant proportion identifying as single (395/411, 96.1%). The age distribution was relatively balanced, with 201/411 (48.9%) participants under the age of 20 years and 210/411 (51.1%) aged 20 or older. Regarding academic standing, 194/411 (47.2%) were first-year students, while 217/411 (52.8%) were enrolled in other academic years

**Table 1.** Characteristics of participants (N=411).

Variable	n (%)
Gender	
Male	109 (26.5)
Female	302 (73.5)
Marital status	
Single	395 (96.1)
Married	16 (3.9)
Age (years)	
<20	201 (48.9)
≥20	210 (51.1)
Education level	
1st year	194 (47.2)
Other years	217 (52.8)

Table 2 presents the distribution of FOMO and SMA scores among participants. The findings indicate that a majority of participants (247/411, 60.1%) exhibited low FOMO scores, while 155/411 (37.7%) fell within the moderate range, and only 9/411 (2.2%) attained high scores. This distribution reflects an overall lower prevalence of FOMO. In contrast, the analysis of SMA scores reveals that 124/411 (30.2%) of participants scored low, 261/411 (63.5%) were categorized within the moderate range, and 26/411 (6.3%) achieved high scores. These results suggest that, although most participants displayed low to moderate levels of FOMO, a notable prevalence of SMA was observed, with a significant proportion reporting moderate to high levels of addiction. Additionally, participants exhibited moderate levels of extraversion, neuroticism, conscientiousness, and openness, while agreeableness recorded the lowest scores.

**Table 2.** Distribution of FOMO,^a^ SMA,^b^ and personality traits scores by frequency and percentage.

Variable	Value
FOMO, n (%)	
Low (≤2.33)	247 (60.1)
Moderate (2.34‐3.67)	155 (37.7)
High (≥3.68)	9 (2.2)
SMA, n (%)	
Low (≤2.33)	124 (30.2)
Moderate (2.34‐3.67)	261 (63.5)
High (≥3.68)	26 (6.3)
Personality traits, mean (SD)	
Extraversion	3.1 (0.5)
Agreeableness	2.0 (0.5)
Conscientiousness	2.8 (0.6)
Neuroticism	3.1 (0.8)
Openness	2.6 (0.5)

a FOMO: fear of missing out.

b SMA: social media addiction.

The analysis demonstrates substantial gender disparities in both FOMO and SMA. Male participants exhibited elevated levels of FOMO (mean 2.43, SD 0.61) in comparison to female participants (mean 2.16, SD 0.63), with a t statistic of 3.86 (*P*<.001). Likewise, males reported higher levels of SMA (mean 2.82, SD 0.62) than their female counterparts (mean 2.64, SD 0.65), with a *t* value of 2.51 (*P*=.013). Nevertheless, the analysis did not identify any significant differences in FOMO or SMA as a function of age or educational attainment (Table 3).

**Table 3.** Group differences in fear of missing out and social media addiction by demographic variables.

Variables	FOMO			SMA		
	Mean (SD)	*t* test (*df*)^a^	*P* value	Mean (SD)	*t* test (*df*)^a^	*P* value
Gender		3.86 (409)	<.001		2.51 (409)	.013
Male	2.43 (0.61)			2.82 (0.62)		
Female	2.16 (0.63)			2.64 (0.65)		
Age (years)		−1.53 (409)	.128		−1.30 (409)	.193
<20	2.18 (0.63)			2.64 (0.63)		
≥20	2.28 (0.64)			2.73 (0.65)		
Education level		−0.93 (409)	.351		−0.24 (409)	.810
1st year	2.20 (0.62)			2.68 (0.63)		
Other years	2.26 (0.65)			2.69 (0.66)		

a Two-tailed

Table 4 delineates notable gender differences in specific personality traits. Male participants exhibited significantly higher scores than female participants in both neuroticism and openness. These findings indicate that gender may influence the development of certain personality dimensions among nursing students. Additionally, no significant differences were detected across age groups or educational levels with regard to any of the assessed personality traits.

**Table 4.** Group differences in personality traits by gender, marital status, age, and education level.

Variables or categories		Gender		Age (years)		Education level	
		Male	Female	<20	≥20	1st year	Other years
Extraversion	Mean (SD)	3.12 (0.46)	3.06 (0.47)	3.06 (0.47)	3.08 (0.47)	3.05 (0.47)	3.09 (0.47)
	*t* test (*df*)^a^	1.23 (409)		−0.45 (409)		−0.84 (409)	
	*P* value	.22		.656		.401	
Agreeableness	Mean (SD)	2.04 (0.47)	1.98 (0.47)	2.02 (0.49)	1.97 (0.44)	2.01 (0.49)	1.99 (0.45)
	*t* test (*df*)^a^	1.06 (409)		1.13 (409)		0.39 (409)	
	*P* value	.29		.26		.696	
Conscientiousness	Mean (SD)	2.8 (0.47)	2.73 (0.57)	2.77 (0.56)	2.73 (0.54)	2.79 (0.57)	2.72 (0.53)
	*t* test (*df*)	1.2 *(409)*		0.85 *(409)*		1.22 *(409)*	
	*P* value	.232		.397		.224	
Neuroticism	Mean (SD)	3.25 (0.68)	2.98 (0.76)	2.99 (0.74)	3.11 (0.75)	2.98 (0.76)	3.11 (0.73)
	*t* test (*df*)	3.3 *(409)*		−1.57 *(409)*		−1.66 *(409)*	
	*P* value	.001		.111		.098	
Openness	Mean (SD)	2.67 (0.48)	2.56 (0.51)	2.59 (0.50)	2.58 (0.51)	2.58 (0.51)	2.6 (0.50)
	*t* test (*df*)	1.98 *(409)*		0.12 *(409)*		−0.39 *(409)*	
	*P* value	.048		.908		.696	

a Two tailed

Table 5 revealed the correlations among FOMO, SMA, and various personality traits. FOMO exhibited a positive correlation with conscientiousness, agreeableness, and SMA, suggesting that individuals displaying elevated levels of FOMO tend to be more conscientious, agreeable, and report increased social media usage. Conversely, FOMO demonstrated a negative correlation with neuroticism. SMA displayed positive correlations with conscientiousness, agreeableness, openness, and extraversion, indicating that these personality traits are associated with heightened levels of SMA. In contrast, neuroticism was negatively correlated with SMA, suggesting that individuals with higher levels of neuroticism reported diminished addiction to social media.

**Table 5.** Correlation analysis between fear of missing out, social media addiction, and personality traits.

Variables	Statistical test	Extraversion	Agreeableness	Conscientiousness	Neuroticism	Openness	SMA
FOMO	*r^a^*	−0.076	0.137	0.261	−0.237	0.077	0.378
	*P* value	.122	.005	<.001	<.001	.117	<.001
SMA	*r*	0.116	0.308	0.500	−0.389	0.160	1
	*P* value	.018	<.001	<.001	<.001	<.001	–^b^

a r: Pearson Correlation

b not applicable

Table 6 demonstrates that the regression model significantly predicts SMA, accounting for 35.8% of its variance (R²=0.358, *P*<.001). Conscientiousness and FOMO emerged as robust positive predictors, whereas neuroticism and female gender were correlated with lower levels of addiction. Notably, conscientiousness exhibited the most substantial effect among all predictors.

**Table 6.** Multiple stepwise regression analysis^a^ of predictors of social media addiction.

Predictor	B	SE B	β	*t* test *(df)^b^*	*P* value	95% CI for B
Constant	1.738	0.249	—	6.977 (4, 406)	<.001	1.248 to 2.227
Conscientiousness	0.421	0.051	.357	8.176 (4, 406)	<.001	0.320 to 0.522
Fear of missing out	0.217	0.043	.213	4.996 (4, 406)	<.001	0.131 to 0.302
Neuroticism	−0.193	0.038	−.223	−5.022 (4, 406)	<.001	−0.268 to −0.117
Gender (female)	−0.143	0.061	−.098	−2.349 (4, 406)	.019	−0.262 to −0.023

a model summary: R²=0.358, Adjusted R²=0.352, F (4, 410)=56.622, *P*<.001

b Two tailed

## Discussion

### Principal Findings and Comparison With Previous Works

The purpose of this study was to investigate the connection between personality traits, FOMO, and SMA in university students, with a specific focus on identifying the main predictors of SMA. The majority of participants had low FOMO scores, whereas SMA scores followed a different pattern, with a larger proportion of participants scoring in the moderate range. In terms of gender differences, male participants exhibited higher levels of both FOMO and SMA compared to female participants. Additionally, male participants scored higher in neuroticism and openness. Regression analysis revealed that both conscientiousness and FOMO positively predicted SMA, while neuroticism and being female were associated with lower levels of addiction.

The results of this study reveal considerable divergence between nursing students’ SMA and FOMO scores. Although most nursing students had low FOMO scores, implying that they do not excessively worry about missing out on things online, more participants had moderate SMA scores. This mirrors findings in other countries. For instance, Turkish nursing students reported moderate degrees of SMA, with lower degrees of FOMO [24]. Another Turkish study did not find a significant correlation between the use of smartphones, FOMO, and care behavior, which implies that FOMO may not always be highly associated with SMA among nursing students [25]. In contrast, in China [26] and in Egypt [27], FOMO was significantly linked to increased SMA, especially among university students, opposite to the present findings. The study’s results can be explained by Saudi cultural norms prioritizing close family and face-to-face interactions, which reduce the emotional impact of FOMO [28]. However, students still engage in social media due to habits, stress relief, and peer pressure, leading to moderate addiction scores [29]. Social media usage is part of life in Saudi Arabia, and though students may not experience extreme FOMO, they may still be affected by the flow of information and social interaction that sites provide. Therefore, future studies are highly recommended to explore the link between FOMO and SMA among nursing students in different cultures.

Additionally, the study reveals gender differences, with male participants exhibiting higher levels of both FOMO and SMA compared to female participants. A previous study has also confirmed a positive correlation between FOMO and SMA. For example, Brailovskaia et al [30] found that, among 745 social media users in Germany, male participants exhibited higher FOMO and addiction, attributing this to their greater need for social validation. However, Kargın et al [28] found no significant gender differences in FOMO and internet addiction among Turkish nursing students, while Li et al [31] reported similar FOMO levels in both sexes in China among university students. These differences could be attributed to cultural and contextual factors, as in Saudi Arabia, where men may experience more pressure to maintain a social image, potentially explaining their higher FOMO and addiction scores compared to women.

Furthermore, the study’s findings indicate that male participants scored higher in neuroticism and openness. Neuroticism is exemplified by emotional instability and heightened sensitivity to stress and, therefore, anxiety. Young male participants, in particular, may have stronger emotional responses to social media use. These emotional responses may lead them to seek validation or manage negative emotions through social media, which can lead to addiction. The positive relationship between neuroticism and SMA was reported [32,33]. A study by Tekin and Turhan [32] found that individuals with high neuroticism are more likely to experience negative emotions on social media, contributing to compulsive use driven by envy and jealousy. Their tendency to overanalyze and engage in social comparison further increases the desire for social media use, raising the risk of addiction. Additionally, male participants with high openness are more inclined to explore new experiences and engage with dynamic content on social media, driven by curiosity and a desire for trends and self-expression [3]. This ongoing engagement can contribute to addiction-like behavior. Therefore, further research about gender and personality traits is encouraged.

Regression analysis revealed that both conscientiousness and FOMO positively predicted SMA, while neuroticism and being female were associated with lower levels of addiction. This finding is inconsistent with a meta-analysis study by Rajesh and Bangaiah [34], which found a negative relation between conscientiousness and Facebook addiction. Furthermore, among university students in Mexico, while SMA is positively associated with neuroticism, it is negatively associated with conscientiousness [35]. Additionally, conscientiousness, openness to experience, and agreeableness were identified as negative predictors of Facebook addiction among Turkish university students [36]. Moreover, a meta-analysis found that neuroticism was positively associated with internet addiction, while openness, agreeableness, extraversion, and conscientiousness were negatively associated [37]. However, in the United States, among 337 college students, none of the personality traits were found to have a relationship with addiction on Facebook, Instagram, and Snapchat [38]. The current study result can be explained by that conscientious nursing students would use social media to stay organized or updated and thus use it more regularly, while FOMO makes people stay connected and not miss social life, thus increasing addiction vulnerability. Nevertheless, neurotic nursing students would experience negative feelings through the use of social media, and hence reduce their use and weaken their addiction. The lower addiction rates among female participants could reflect more balanced and responsible use, potentially due to different usage patterns or social norms.

Based on the results of this study, there is a vital implication for nursing practice that needs to be considered. Students should be directed to various social, artistic, and sporting activities that aim to support the use of the internet for the benefit of students, provide effective training in social communication and internet awareness, and reduce excessive time spent in social media environments. Psychiatric nurses working in units where primary health care services are provided are known to have an important role in combating addiction, and are also in a key position in the training that will be provided to students in this area. More research should be conducted to identify factors associated with the severity of FOMO.

Likewise, nursing practitioners and educators should be aware of the degree to which social media use is driven by personality traits and how such use impacts mental health. Low conscientiousness or high neuroticism may predispose students to increase SMA as they seek incentives or prevent adverse effects. Being aware of these tendencies may allow nursing educators to provide better care for vulnerable students. Interventions may include integrating digital well-being into nursing education, teaching students how to balance social media use, and promoting healthy coping strategies for managing FOMO. By addressing these issues, nursing schools can not only improve the overall well-being of their students but also equip future healthcare professionals with the skills they need to manage their mental well-being and be an example to patients they will interact with.

### Limitations

Several limitations should be acknowledged. First, the data were collected using self-reported questions, which may introduce bias. Second, the responses are subject to recall bias. Additionally, the study measured SMA at a specific point in time, without considering other temporal factors that might influence social media use. Furthermore, the use of a convenience sampling technique may limit the diversity of the sample. Finally, data were gathered only from a nursing college at one university, which may limit the generalizability of the findings.

### Conclusion

This study aimed to explore the relationship between personality traits, FOMO, and SMA among Saudi nursing university students. The results indicate that conscientiousness and FOMO are positive predictors of SMA, while neuroticism is negatively associated. Furthermore, male participants tend to have higher levels of both FOMO and SMA than female participants. These findings highlight the influence of personality traits and FOMO on SMA in university students. Thus, strategies that help nursing students in getting the optimum benefits from social media need to be examined and implemented.

## Abbreviations

FOMO: fear of missing out
SMA: social media addiction
